# Toll-like receptor 4: a target for chemoprevention of hepatocellular carcinoma in obesity and steatohepatitis

**DOI:** 10.18632/oncotarget.25685

**Published:** 2018-06-29

**Authors:** Jennifer Nguyen, Jingjing Jiao, Kristin Smoot, Gordon P. Watt, Chen Zhao, Xingzhi Song, Heather L. Stevenson, Joseph B. McCormick, Susan P. Fisher-Hoch, Jianhua Zhang, P. Andrew Futreal, Laura Beretta

**Affiliations:** ^1^ Department of Molecular and Cellular Oncology, The University of Texas MD Anderson Cancer Center, Houston, TX, USA; ^2^ University of Texas Health Science Center at Houston, School of Public Health in Brownsville, Brownsville, TX, USA; ^3^ Department of Genomic Medicine, Division of Cancer Medicine, The University of Texas MD Anderson Cancer Center, Houston, TX, USA; ^4^ Department of Pathology, The University of Texas Medical Branch, Galveston, TX, USA

**Keywords:** toll-like receptor 4, chemoprevention, NAFLD, hepatocellular carcinoma, mouse model

## Abstract

The incidence of hepatocellular carcinoma (HCC) associated with non-alcoholic fatty liver disease (NAFLD) is rapidly increasing. We aimed to elucidate the genetic basis of NAFLD-associated HCC and identify candidate targets for chemoprevention. Twenty HCC tumors, distant liver and matched tails from mice with hepatocyte-deletion of Pten (Hep*Pten*^-^) were subjected to whole-exome sequencing. A total of 162 genes with somatic non-synonymous single nucleotide variants or exonic small insertions and deletions in tumors were identified. Ingenuity Pathway Analysis of these 162 genes, further identified Toll-like receptor (TLR) 4, a key mediator of proinflammatory responses, and resatorvid, a TLR4 inhibitor, as the main causal networks of this dataset. Resatorvid treatment strongly prevented HCC development in these mice (*p* < 0.001). Remarkably, HCC patients with high tumoral TLR4 mRNA expression were more likely to be diagnosed with NAFLD and obese. TLR4 mRNA expression positively correlated with IL-6 and IL-10 mRNA expression in HCC tumors and the correlation was stronger in obese HCC patients. We have identified tumor mutation signatures and associated causal networks in NAFLD-associated HCC in Hep*Pten*^-^ mice and further demonstrated the important role of TLR4 in promoting HCC development. This study also identified IL-6 and IL-10 as markers of TLR4 activation in HCC and subjects with NAFLD and obesity as the target population who would benefit from TLR4 inhibition treatment for HCC chemoprevention.

## INTRODUCTION

Hepatocellular carcinoma (HCC) is the second leading cause of cancer-related mortality worldwide [1]. In the United States, HCC incidence and mortality rates are rapidly increasing, in part due to the epidemics of obesity and diabetes, leading to non-alcoholic fatty liver disease (NAFLD) [2, 3]. NAFLD is a spectrum of diseases that begins with simple steatosis and eventually progresses to steatohepatitis (NASH), leading to cirrhosis and HCC [4]. Most patients with HCC are diagnosed at an advanced stage when limited treatment options are available, often due to the presence of cirrhosis and poor liver function. Hence, the development of novel therapeutic options for the treatment of NASH and prevention of HCC is urgently needed [5, 6].

In this study, we aimed to elucidate the genetic basis of NAFLD-associated HCC and identify candidate targets for chemoprevention. To that end, we performed whole-exome sequencing on tumors and used Ingenuity Pathway Analysis (IPA) to identify candidate genetic drivers of HCC in a mouse model of NASH-related HCC. We further validated the main candidate target in HCC prevention studies *in vivo* and characterized the target's expression in human HCCs. Mouse models are extensively used and readily accessible sources of invaluable information when developing therapeutic strategies for human diseases. Mouse models for target discovery should reproduce the natural pathogenesis of the human disease of interest. We used mice with hepatocyte-specific Pten deletion (Hep*Pten-*). Hep*Pten-* mice develop steatosis, liver fibrosis, NASH and HCC [7, 8]. This model most closely resembles both the histopathology of and the molecular changes associated with human NASH and HCC [9]. In addition, human NASH and HCC are characterized by PTEN mutations, inhibition of PTEN expression, or loss of PTEN function [10, 11].

## RESULTS

### Whole-exome sequencing of HCCs and Ingenuity Pathway Analysis identified TLR4 as a candidate target for HCC prevention in HepPten^-^ mice

To elucidate the genetic basis of NASH-associated HCC and identify candidate targets for chemoprevention, 20 HCC tumors, distant liver and matched tails collected from ten male mice with hepatocyte-deletion of Pten (Hep*Pten*^-^) were subjected to whole-exome sequencing (WES). Somatic single nucleotide variants (SNVs) and small insertions and deletions (indels) were identified by comparing tumor or distant nontumor liver samples to tails. A total of 90 somatic non-synonymous single nucleotide variants (SNVs) and 80 exonic small insertions and deletions (indels) covering 162 genes were identified in tumors. The co-mutation data of each HCC tumor is shown in Supplementary Table 1. Among these 162 genes, 8 were also mutated in the adjacent non tumoral liver. The number of mutations detected in each tumor ranged from 2 to 25 with an average of 10 mutations per tumor. The highest frequency (3 out of 20 tumors) was observed for a frameshift insertion mutation in the FK506 binding protein 7, *Fkbp7* (A129fs). Genes mutated in 2 out of the 20 tumors were *Atp5g3, Cdh7, Dennd2a, Esco2, Fndc3a, Gm1995, Gopc, Grb10, Mdc1, Nop58, Polr3c, Senp6, Serpinb3a, Serpinb3b, Serpinb3d, Spice1, Stxbp3a, Xylt2,* and *Zzef1*. All other genes were found mutated in a single tumor and included known drivers of hepatocarcinogenesis such as *Birc6, Hras, and Kmt2a*. Among these 162 genes, 119 were found mutated in HCC in TCGA HCC database, representing 64% of all HCCs.

**Figure 1:**
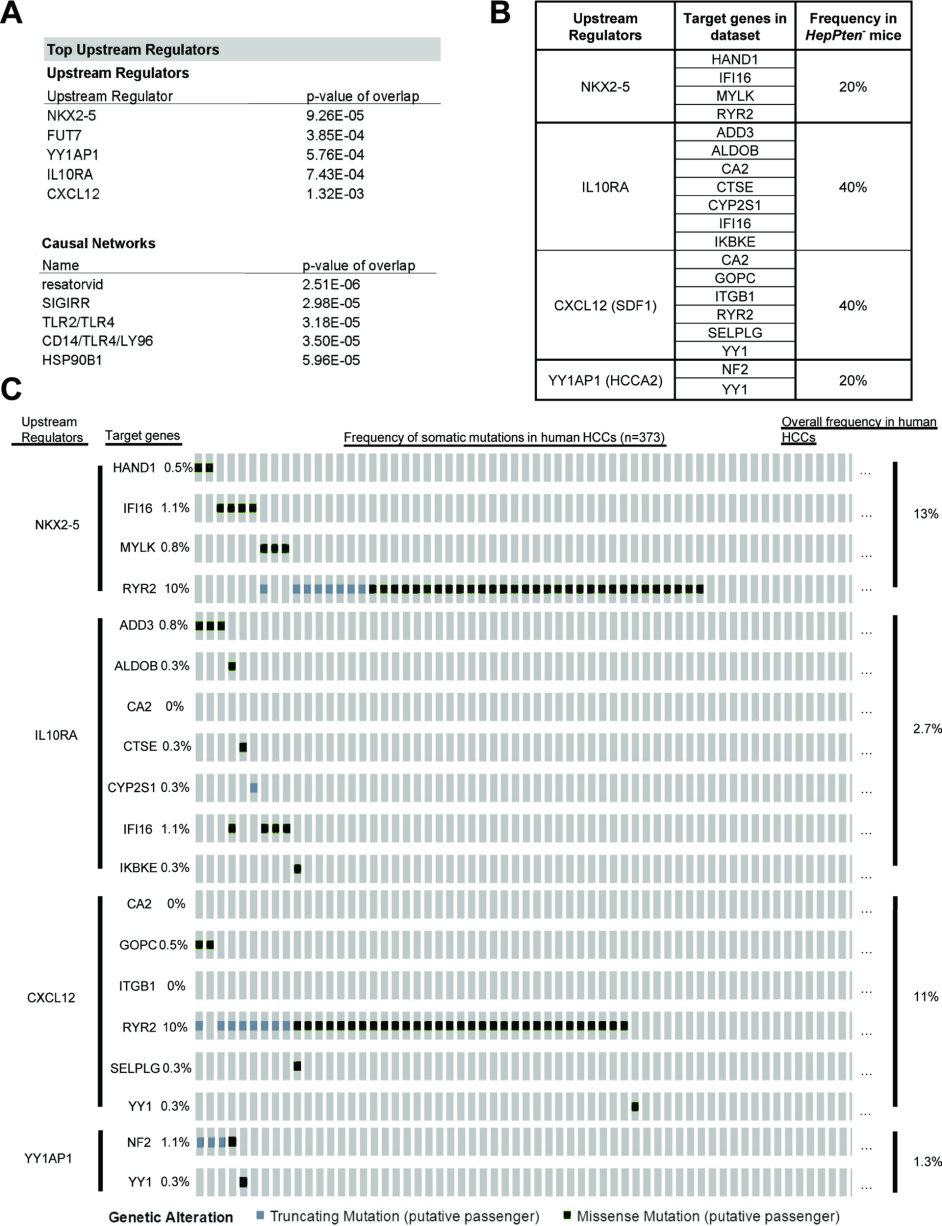
WES and IPA analysis reveals relevant biological targets of HCC in Hep*Pten^-^* mice (**A**) IPA results of the 162 genes identified mutated in HCC tumors in Hep*Pten^-^* mice. Core analysis on IPA identified top upstream regulators and causal networks for the dataset. (**B**) Percentage of Hep*Pten^-^* mice that carried a mutation in at least one of the target genes for each identified upstream regulator. (**C**) Percentage of HCC patients in TCGA that carried a mutation in at least one of the target genes for each identified upstream regulator.

To identify potential therapeutic targets, the list of the 162 genes was uploaded onto Ingenuity Pathway Analysis (IPA) software for upstream and causal network analysis. Core Analysis by IPA identified 5 top upstream regulators that directly target genes from the dataset: NK2 homeobox 5 (NKX2-5) (*p* = 9.26 × 10^–5^), fucosyltransferase 7 (FUT7) (*p* = 3.85 × 10^–4^), Yin-Yang 1 associated protein 1 (YY1AP1) (*p* = 5.76 × 10^–4^), interleukin 10 receptor alpha (IL10RA) (*p* = 7.43 × 10^–4^) and stromal-derived factor 1 (CXCL12) (*p* = 1.32 × 10^–3^) (Figure 1A). To further evaluate the relevance of the identified top upstream regulators, we sought to determine the mutation distribution of target genes in each network in the Hep*Pten^-^* model as well as in human HCCs using the cBioPortal-TCGA HCC database (Figure 1B–1C). For each upstream regulator, 2 to 8 target genes were found mutated in Hep*Pten*^-^ tumors. These include *Hand1, Ifi16, Mylk,* and *Ryr2* regulated by upstream *Nkx2-5*; *Add3, Aldob, Ca2, Ctse, Cyp2s1, Ifi16,* and *Ikbke* regulated by upstream *Il10ra*; *Ca2, Gopc, Itgb1, Ryr2, Selplg,* and *Yy1* regulated by upstream *Cxcl12 (SDF1)*; and *Nf2* and *Yy1* regulated by upstream *Yy1ap1 (Hcca2)* (Figure 1B). These 4 altered networks were detected in 20–40% of Hep*Pten*^-^ mice and in 1.3–13% of human HCCs (Figure 1B–1C).

Core Analysis by IPA also identified significant causal networks. The top four causal networks were all found to be closely associated with toll-like receptor (TLR) 4 (Figure 1A). Resatorvid (*p* = 2.51 × 10^–6^) is a small molecule that specifically inhibits TLR4 by binding to Cys^747^ in the intracellular TIR domain of TLR4 [12]. Single immunoglobulin interleukin-1 receptor related molecule (SIGIRR) (*p* = 2.98 × 10^–5^) inhibits TLR4 signaling through interaction with TLR4, MD2, MyD88, and TIRAP [13]. TLR2/TLR4 (3.18 × 10^–5^) are both implicated in recognizing various bacterial cell wall components and both signal an inflammatory response through MyD88 [14]. CD14 and LY96 (*p* = 3.50 × 10^–5^) are adaptor proteins that assist in maneuvering ligands to TLR4 [15]. Based on these results, TLR4 was identified as the main driver of HCC development in Hep*Pten*^-^ mice and resatorvid as the main candidate therapeutic drug for the prevention of NASH-associated HCC.

### Resatorvid prevented HCC development in HepPten^-^ mice

**Figure 2:**
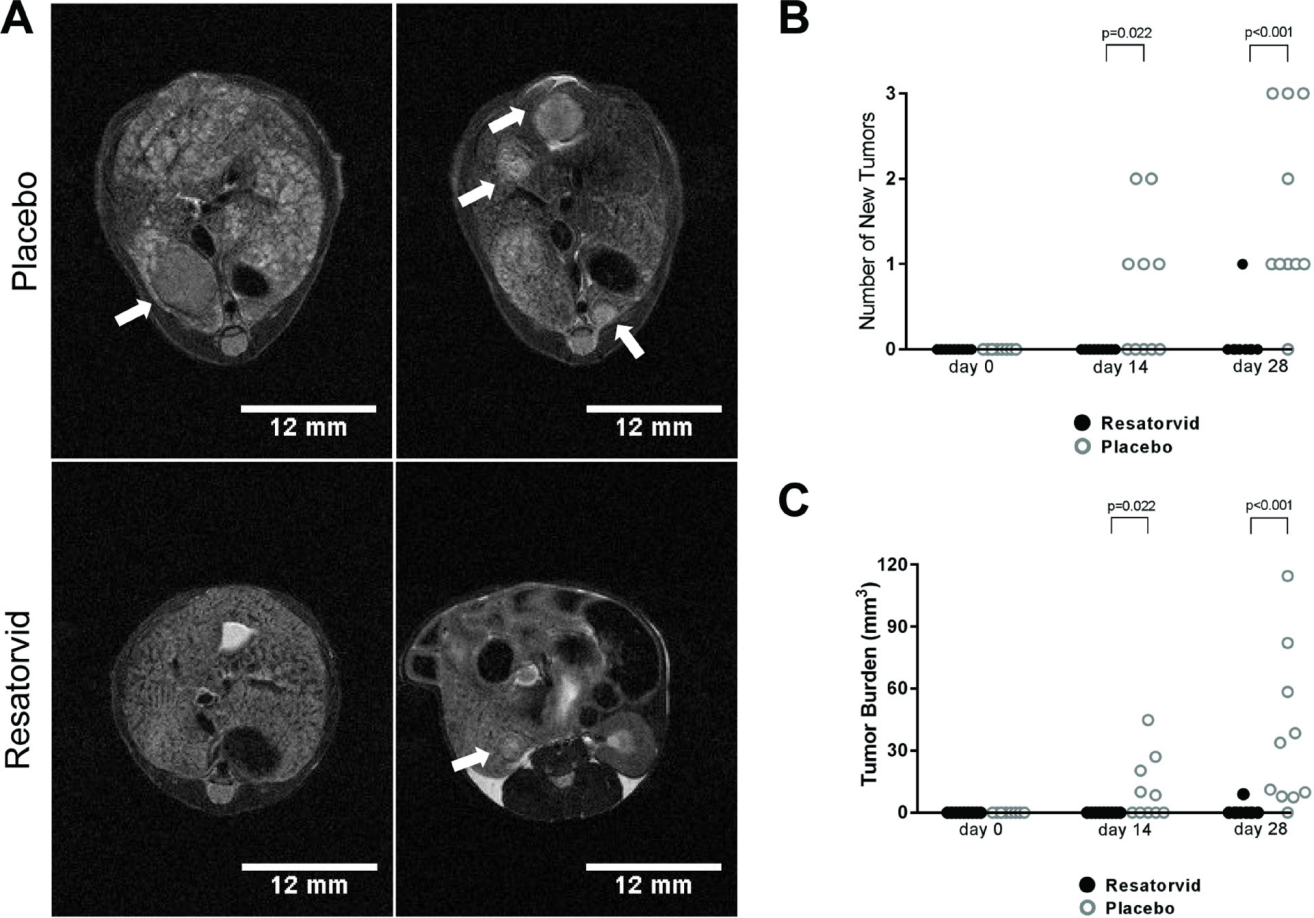
Resatorvid prevents HCC development in Hep*Pten^-^* mice (**A**) Livers of placebo- and resatorvid-treated mice were imaged by MRI to monitor for tumor development and measure tumor size. Representative tumors detected by MRI at the end of treatment are indicated by white arrows. (**B**) Number of new tumors with volumes ≥ 7.5 mm^3^ in placebo-treated and resatorvid-treated mice as detected by MRI at day 14 and day 28. Data are presented as the number of tumors detected in each mouse (unpaired Mann–Whitney test). (**C**) Tumor burden in placebo-treated and resatorvid-treated mice detected by MRI and measured with ImageJ at day 14 and day 28. Data are presented as the total tumor burden in each mouse (unpaired Mann–Whitney test).

We then investigated whether resatorvid treatment could prevent HCC development in Hep*Pten*^-^ mice. Because HCC develop in male Hep*Pten*^-^ mice between 8 and 9 months of age, we selected for the study nineteen 8-month old male mice with no tumors as confirmed by MR imaging (MRI) and separated them into a placebo group (*n* = 10) and a resatorvid-treated group (*n* = 9). Hep*Pten^-^* were treated with resatorvid daily for 28 days with intraperitoneal injections of 10 mg/kg and mice were imaged by MRI at day 0, day 14 and day 28. Representative MRI images of tumors detected at day 28 are shown in Figure 2A. At day 14, no tumor could be detected in the resatorvid-treated mice while 5 of the 10 placebo mice developed 1 or 2 tumors (*p* = 0.022). At day 28, up to 3 tumors were detected in 9 out of 10 placebo mice while in the resatorvid-treated group, only 1 mouse had a detectable small tumor by MRI (*p <* 0.001) (Figure 2A–2B). The average tumor burden in placebo mice was significantly greater than in resatorvid-treated mice at day 14 with 11.1 ± 4.8 mm^3^ (8.4–44.8 mm^3^) vs. 0.0 ± 0.0 mm^3^, respectively (*p* = 0.022); and at day 28 with 36.4 ± 12.0 mm^3^ (7.5–114.6 mm^3^) vs. 1.0 ± 1.0 mm^3^, respectively (*p <* 0.001) (Figure 2C).

### Effects of resatorvid treatment on liver steatosis and fibrosis in HepPten^-^ mice

**Figure 3:**
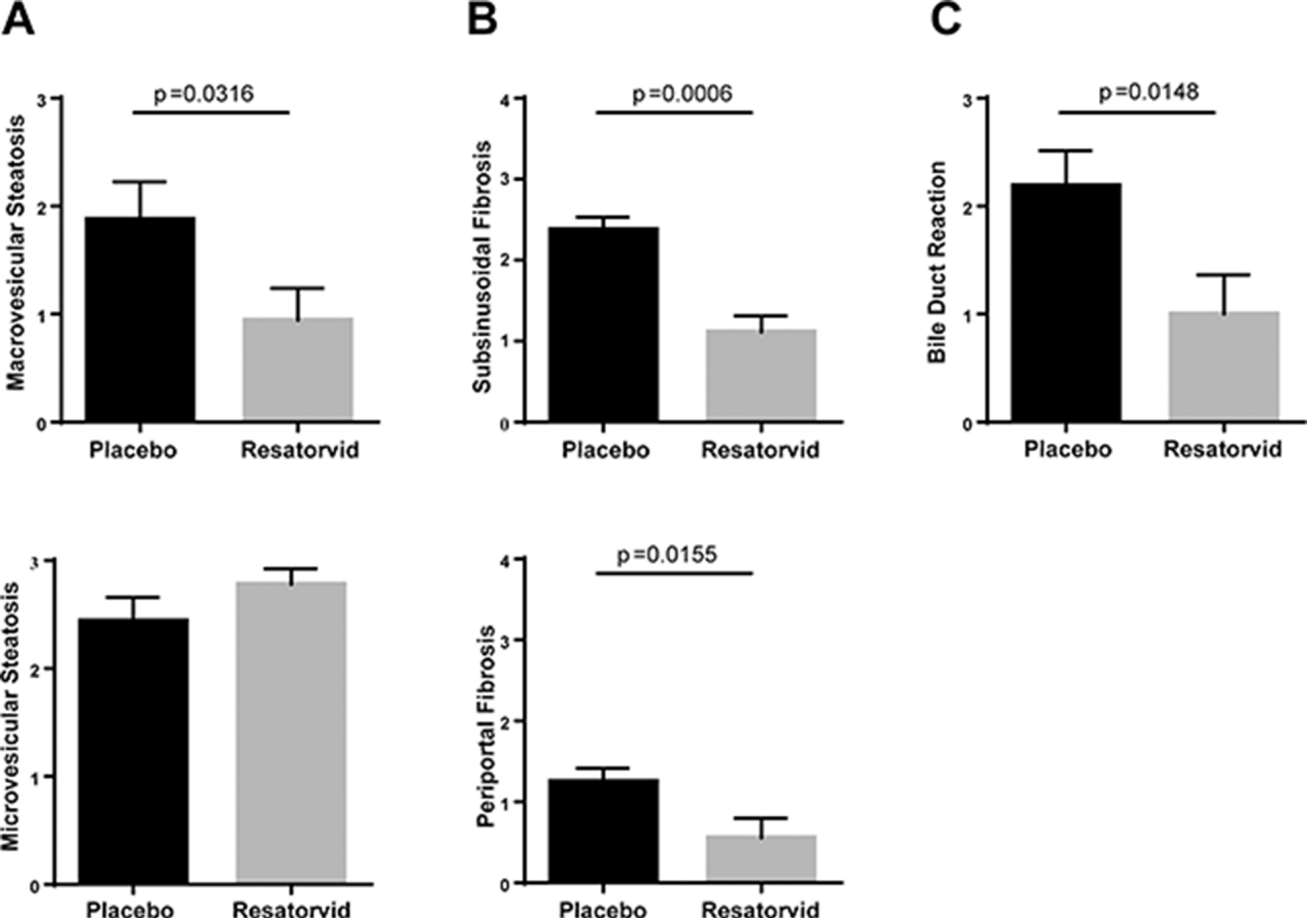
Effects of resatorvid on steatosis and liver fibrosis in Hep*Pten*^-^ mice Livers of placebo and resatorvid-treated mice were analyzed for histopathological features associated with NASH. (**A**) Macrovesicular steatosis was graded on a 0 to 3 scale (0 for <5%, 1 for 5–33%, 2 for 34–66%, and 3 for >66%). (**B**) Subsinusoidal and periportal fibrosis were examined and scored using a scoring system of 0 to 4. (**C**) Bile-duct reaction was assessed based on the number of lesions observed [0 for absent, 1 for 1–4 lesions (focal), 2 for 5–10 lesions (frequent), and 3 for >10 lesions (diffuse)].

Since *HepPten-* mice develop NASH prior to HCC, we also evaluated the effect of resatorvid treatment on this underlying liver pathology. To that end, a liver pathologist analyzed the histology of the liver of the treated mice, blinded to the treatment group. Representative H&E and Masson Trichrome's staining images are shown in Supplementary Figure 1. Compared to resatorvid-treated mice, placebo-treated mice had significantly more macrovesicular steatosis and more steatosis overall, which was often panlobular (i.e., extending from portal tracts to central veins); the resatorvid-treated mice only had mild perivenular (zone 3) microvesicular steatosis. Treatment with resatorvid resulted in a significant decrease in macrovesicular steatosis from a score of 1.9 ± 0.4 to a score of 0.9 ± 0.3 (*p* = 0.031) (Figure 3A). No effect on microvesicular steatosis scores was observed. In addition, the placebo-treated group had more prominent bile ductular reactions and increased periportal, subsinusoidal, and perivenular fibrosis. The bile duct lesions often contained scattered inflammatory cells with surrounding fibrosis. Both subsinusoidal fibrosis and periportal fibrosis were significantly reduced upon resatorvid treatment from a score of 2.4 ± 0.2 to 1.1 ± 0.2 (*p <* 0.001) and from a score of 1.3 ± 0.2 to 0.6 ± 0.2 (*p* = 0.015), respectively (Figure 3B). Bile-ductular reaction was also strongly reduced upon resatorvid treatment (*p* = 0.014) (Figure 3C). No significant effect was observed on hepatocyte ballooning degeneration and inflammation.

### Demographic and clinical characteristics of HCC patients with high tumoral TLR4 mRNA expression

To characterize HCC patients with elevated TLR4 mRNA gene expression in tumors, we retrieved using cBioPortal, clinical, demographic, and mRNA expression data from 363 HCC patients analyzed in The Cancer Genome Atlas (TCGA) (Supplementary Table 2). HCC patients were separated into quartiles, dependent upon respective TLR4 mRNA expression. Groups were dichotomized into “low” versus “high” expression. We defined high TLR4 mRNA expression as quartile Q4 (*n* = 91) and low TLR4 expression as quartiles Q1–Q3 (*n* = 272). The demographic and clinical characteristics of HCC patients with elevated TLR4 mRNA expression in tumors are presented in Table 1. After adjustment to age and sex, high TLR4 mRNA expression was strongly associated with NAFLD (AOR = 2.73, 95% CI = 1.09–6.85, *p* = 0.032). High TLR4 mRNA expression was also associated with obesity (adjusted Odds Ratio (AOR) = 2.03, 95% CI = 1.13–3.63, *p* = 0.017). The high TLR4 expression group was less likely to be HBV positive (AOR = 0.47, 95% CI = 0.26–0.88, *p* = 0.017) or to have alpha-feto protein (AFP) blood levels above 20 ng/ml (AOR = 0.56, 95% CI = 0.31–1.01, *p* = 0.055). Finally, we did not find any association with gender, age, HCV, alcohol consumption, presence of cirrhosis or family history of cancer.

**Table 1:** Demographic and clinical variables in 363 HCC patients by tumoral TLR4 mRNA expression

	Low TLR4 (Q1-Q3)	High TLR4 (Q4)	*P*	Adjusted OR	*P*
**TLR4 mRNA Expression** (*n* = 363)	152.9 (4.1)*–150.1*	552.0 (29.6)*–452.6*			
**Male** (*n* = 363)	181 (66.5%)	63 (69.2%)	0.6366	1.13 (0.68–1.89)	0.6418
**Race, Ethnicity** (*n* = 344)			0.4965		0.503
Asian	122 (47.5%)	34 (39.1%)		REF	
White, Non-Hispanic	111 (43.2%)	44 (50.6%)		1.66 (0.94–2.91)	0.0784
White, Hispanic	10 (3.9%)	5 (5.7%)		1.89 (0.60–5.95)	0.2785
Other	14 (5.4%)	4 (4.6%)		1.04 (0.32–3.39)	0.9459
**Age (y)** (*n* = 363)	59.59 (0.80)*–61.0*	59.43 (1.38)*–61.0*	0.9185	1.0 (0.98–1.02)	0.9527
**Family History of Cancer** (*n* = 312)	77 (33.9%)	32 (37.6%)	0.539	1.21 (0.71–2.07)	0.477
**BMI** (*n* = 329)	25.4 (0.4)*–24.3*	26.7 (0.8)*–24.7*	0.1037	1.04 (0.99–1.08)	0.0922
**NAFLD** (*n* = 344)	11 (4.2%)	9 (10.7%)	0.0329	2.73 (1.09–6.85)	0.0327
**Obese (BMI ≥ 30)** (*n* = 329)	41 (16.9%)	25 (28.7%)	0.0198	2.03 (1.13–3.63)	0.0173
**HBV** (*n* = 344)	86 (33.1%)	17 (20.2%)	0.0272	0.47 (0.26–0.88)	0.0173
**HCV** (*n* = 344)	43 (16.5%)	13 (15.5%)	0.8187	0.90 (0.46–1.78)	0.7591
**Alcohol Etiology** (*n* = 344)	87 (33.5%)	28 (33.3%)	0.9827	0.94 (0.54–1.63)	0.8112
**Cirrhosis** (*n* = 282)	67 (32.4%)	28 (37.3%)	0.436	1.24 (0.71–2.17)	0.4437
**Fibrosis** (*n* = 248)	124 (66.3%)	37 (60.7%)	0.4222	0.78 (0.42–1.45)	0.4238
**Fibrosis Ishak Score** (*n* = 161)			0.0545		0.0503
1, 2	30 (24.2%)	5 (13.5%)		REF	
3, 4	27 (21.8%)	4 (10.8%)		0.93 (0.23–3.88)	0.9246
5, 6	67 (54.0%)	28 (75.7%)		2.78 (0.96–8.05)	0.0596
**AFP** (ng/mL) (*n* = 273)	18202 (9948)*–23*	877.0 (644.4)*–10*	0.1911	1.00 (1.00–1.00)	0.2053
**AFP ≥ 20 ng/mL** (*n* = 273)	107 (51.0%)	23 (36.5%)	0.0456	0.56 (0.31–1.01)	0.0555

Data are presented as mean (SEM)-*median* or as frequency (%). BMI, body mass index; NAFLD, non-alcoholic fatty liver disease; HBV, hepatitis B virus; HCV, hepatitis C virus.

### Correlation analysis of TLR4, IL-6 and IL-10 mRNA expression and factors associated with high IL-6 or IL-10 mRNA expression in HCC tumors

**Figure 4:**
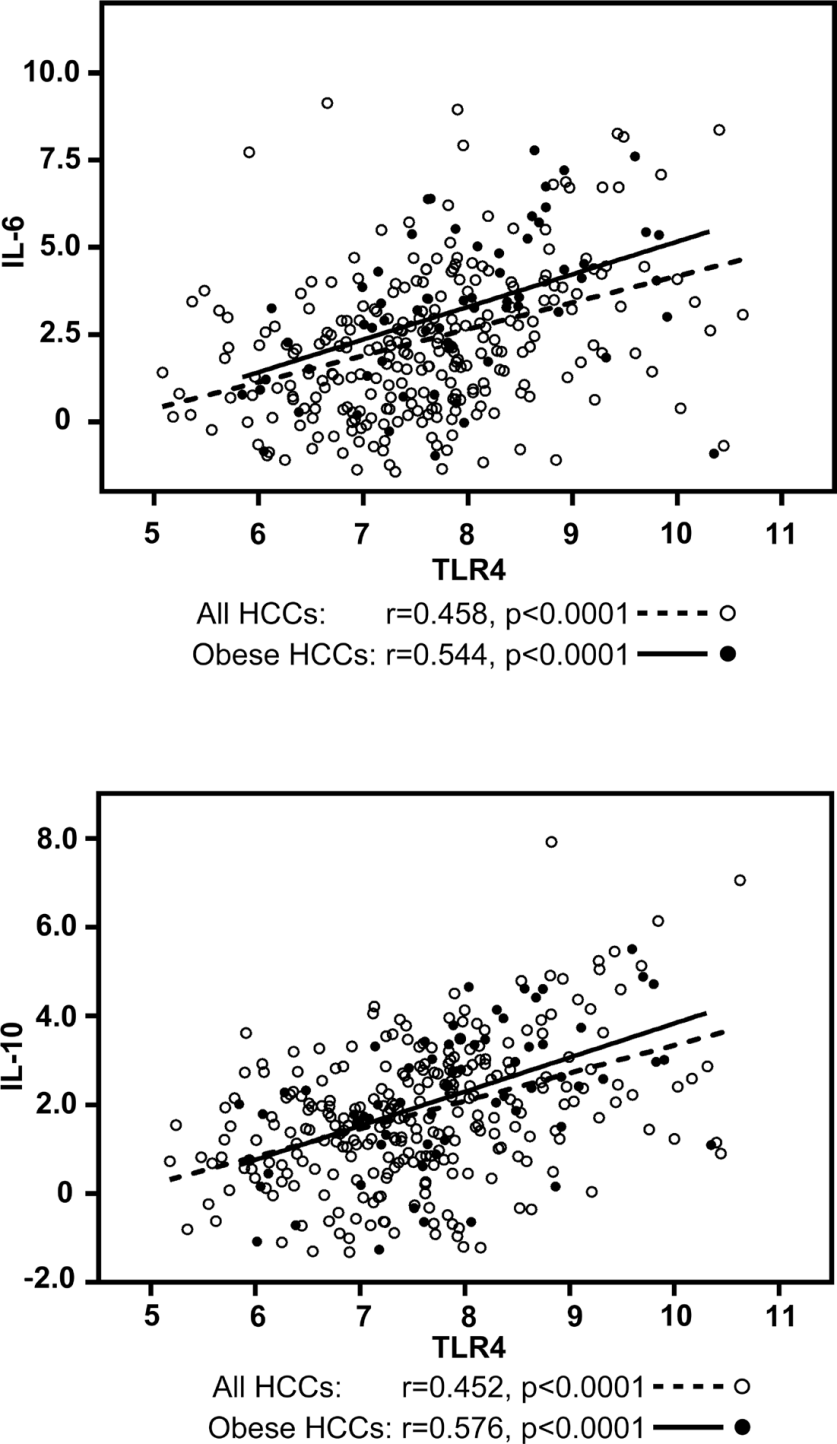
Correlation analysis between TLR4 and IL-6 / IL-10 mRNA expression in HCC tumors in all HCC patients and in obese HCC patients Correlation was evaluated using Spearman rank-order correlation coefficient. Graphs are represented using Log2 values in all HCCs (open dots and dotted lines) and in obese HCCs (dark dots and solid lines).

To identify companion biomarkers of TLR4 activation in HCC, we performed correlation analyses of tumoral mRNA expression between TLR4 and known TLR4-associated cytokines, IL-6 and IL-10, using the same HCC gene expression dataset from TCGA. TLR4 mRNA expression strongly correlated with IL-6 mRNA expression (*r* = 0.458, *p <* 0.0001) and IL-10 mRNA expression (*r* = 0.452, *p <* 0.0001) (Figure 4). The correlation was even stronger in obese HCC patients (*n* = 66) with *r* = 0.544, *p <* 0.0001 for IL-6 and *r* = 0.576, *p <* 0.0001 for IL-10 (Figure 4). HCC patients were again separated into quartiles, by IL-6 or by IL-10 mRNA expression. The demographic and clinical characteristics of HCC patients with elevated IL-6 mRNA expression in tumors are presented in Supplementary Table 3 and summarized in Table 2A. After adjustment for age and sex, high IL-6 mRNA expression was also associated with obesity (AOR = 3.11, 95% CI = 1.74–5.56, *p* = 0.0001) and NAFLD (AOR = 2.59, 95% CI = 1.03–6.52, *p* = 0.042) and inversely associated with HBV (AOR = 0.34, 95% CI = 0.17–0.65, *p* = 0.0013). In addition and in contrast to TLR4, a strong association was found with HCV (AOR = 2.98, 95% CI = 1.61–5.50, *p* = 0.0005). The frequency of HCV was 12.4% in HCC patients with low IL-6 mRNA gene expression but reached 27.9% in the high IL-6 expression group. As for TLR4, we did not find any association with gender, age, alcohol etiology, cirrhosis or family history of cancer. The only significant association found with high IL-10 mRNA expression in HCC tumors was obesity (AOR = 2.34, 95% CI = 1.30–4.23, *p* = 0.004) (Supplementary Table 4 and Table 2B). The frequency of obese individuals in low and high IL-10 mRNA gene expression was 16.5% and 30.9% respectively.

**Table 2:** Demographic and clinical variables in 363 HCC patients by (A) tumoral IL-6 and (B) tumoral IL-10 mRNA expression

A					
	**Low IL-6 (Q1–Q3)**	**High IL-6 (Q4)**	***P***	**Adjusted OR**	***P***
**IL6 mRNA Expression** (*n* = 363)	3.19 (0.18)*–2.22*	63.00 (10.24)*–22.01*			
**BMI** (*n* = 329)	25.1 (0.4)*–24.0*	27.7 (0.8)*–26.3*	0.0019	1.07 (1.03–1.11)	0.0018
**NAFLD** (*n* = 344)	11 (4.3%)	9 (10.5%)	0.0392	2.59 (1.03–6.52)	0.0427
**Obese (BMI ≥ 30)** (*n* = 329)	37 (15.0%)	29 (35.4%)	0.0001	3.11 (1.74–5.56)	0.0001
**HBV** (*n* = 344)	90 (34.9%)	13 (15.1%)	0.0008	0.34 (0.17–0.65)	0.0013
**HCV** (*n* = 344)	32 (12.4%)	24 (27.9%)	0.0010	2.98 (1.61–5.50)	0.0005
**Alcohol Etiology** (*n* = 344)	86 (33.3%)	29 (33.7%)	0.9474	1.14 (0.66–1.99)	0.6418
**B**					
	**Low IL10 (Q1*–*Q3)**	**High IL10 (Q4)**	***P***	**Adjusted OR**	***P***
**IL-10 mRNA Expression** (*n* = 363)	2.47 (0.11)*–2.32*	18.55 (2.99)*–11.05*			
**BMI** (*n* = 329)	25.3 (0.4)*–24.2*	27.1 (0.9)*–25.0*	0.0242	1.05 (1.01–1.10)	0.0153
**NAFLD** (*n* = 344)	14 (5.4%)	6 (7.0%)	0.5956	1.33 (0.49–3.59)	0.5726
**Obese (BMI ≥ 30)** (*n* = 329)	41 (16.5%)	25 (30.9%)	0.0059	2.34 (1.30–4.23)	0.0046
**HBV** (*n* = 344)	83 (32.2%)	20 (23.3%)	0.1198	0.59 (0.33–1.06)	0.0783
**HCV** (*n* = 344)	41 (15.95)	15 (17.4%)	0.7360	1.14 (0.59–2.19)	0.7044
**Alcohol Etiology** (*n* = 344)	80 (31.0%)	35 (40.7%)	0.1002	1.69 (0.98–2.92)	0.0593

Data are presented as mean (SEM)-*median* or as frequency (%). BMI, body mass index; NAFLD, non-alcoholic fatty liver disease; HBV, hepatitis B virus; HCV, hepatitis C virus.

## DISCUSSION

In this study, we used an unbiased genomic discovery approach in a mouse model of NASH-associated HCC, to identify candidate targets and drugs for HCC prevention. Candidate target and drug identified were toll like receptor 4 (TLR4) and resatorvid, an inhibitor of TLR4. Resatorvid (or TAK-242) was first discovered in 2006, as a suppressor of cytokine production and inhibitor of TLR4 intracellular signaling, while developing novel drugs to treat and/or prevent the septic shock associated with infections caused by Gram-negative bacteria [16, 17]. It was subsequently demonstrated that resatorvid is a selective inhibitor of signaling from the intracellular domain of TLR4, disrupting TLR4's interaction with adaptor molecules [12, 18]. A randomized, double-blind, placebo controlled trial of resatorvid for the treatment of severe sepsis, showed that treatment was well tolerated and resulted in lower mortality rates in patients with both shock and respiratory failure, although not significantly and without suppression of cytokine levels [19]. Our study demonstrated that resatorvid very strongly prevented HCC development in a mouse model of NASH-associated HCC relevant to the human disease.

The role of toll-like receptors TLRs in hepatic inflammation and fibrosis has attracted much attention. In liver, TLR4 is expressed by all parenchymal and non-parenchymal cell types, and contributes to tissue damage, liver fibrosis, NASH, and HCC progression (reviewed in [20–24]). A role for endothelial cell TLR4 in fibrosis-associated angiogenesis in the liver was also proposed [25]. TLR4's key function in liver diseases was further substantiated when associations between single nucleotide polymorphisms (SNPs) of the TLR4 gene in humans and risks of specific diseases, including cirrhosis, were reported. TLR4 D299G and T399I SNPs were reported to be associated with protection from hepatic fibrosis by reducing TLR4-mediated fibrogenic signaling [26]. It was also proposed that TLR4 SNPs could play an important protective role in the development of HCC [27]. Patients with C159T SNP in CD14, a co-receptor of TLR4, have an increased risk of NAFLD development [28]. In Ob/Ob mice, development of steatohepatitis was shown to be dependent on TLR4 [29]. In mice with hepatocyte-deletion of Pten, TLR4 but not TLR2 deficiency suppressed tumor growth as well as hepatic inflammation, in good agreement with the results of our study. The authors suggested that TLR4 on macrophages contributes to the development of steatohepatitis-related HCC in this model [30]. Cooperation between TLR4 signaling and STAT3 in promoting tumor-initiating stem-like cells in mouse liver was recently reported [31]. Interestingly, we showed that a small molecule inhibitor of STAT3 blocked HCC tumor growth, reduced tumor development, and improved NASH in Hep*Pten-* mice, and that these effects were associated with an inhibition of TLR signaling pathways [32]. Future studies should be pursued to identify the TLR-4-expressing cells and mechanisms that mediate the chemopreventive effect of resatorvid. Whether the effects of TLR4 on NASH and HCC development in our model are dependent on the gut microbiome should be further investigated. Indeed, emerging data have recently shown a close association between compositional changes in gut microbiota and the development of NAFLD. Gut microbiota are a source of TLR ligands, and their compositional change can also increase the amount of TLR ligands delivered to the liver. Remarkably, recent studies showed promotion of HCC by the intestinal microbiota and TLR4 [33] and that gut-derived LPS promotes T-cell-mediated hepatitis in mice through TLR4 [34].

To determine who would benefit from TLR4 inhibition treatment for HCC prevention, we characterized using TCGA HCC public datasets, HCC patients with elevated TLR4 mRNA expression as well as HCC patients with elevated mRNA expression of two known cytokines produced by TLR4 activation, IL-6 and IL-10. Among all HCCs, patients with NAFLD and obese patients had the higher tumoral expression of TLR4, IL-6 and IL-10. In addition, TLR4 and IL-6 or IL-10 mRNA expression in tumors were found to strongly correlate in obese HCC patients. Future studies should evaluate the effects of resatorvid in high fat diet induced steatosis and consequent HCC development in mouse models.

Besides TLR4, a number of upstream regulators, potential drivers of HCC in NASH were also identified. NKX2-5 is a transcription factor known to regulate *β-catenin* transcription and with a potential role in HCC development [35]. YY1AP1 was recently identified as an oncogenic driver in EpCam (+) AFP(+) HCC by altering the chromatin landscape and activating stem-like features [36]. IL-10 and associated immune pathway promote a favorable environment for hepatocarcinogenesis thus accelerating HCC progression in NASH [37, 38]. CXCL12 activates CXC chemokine receptor 4 (CXCR4) resulting in liver fibrosis, tumor growth, and HCC metastasis [39–41]. All these genes should be further investigated.

In conclusion, this study highlighted the specific molecular events and signaling pathways in the pathogenesis of NAFLD-associated HCC and identified the important role of TLR4 in promoting HCC development in the context of NASH. This study demonstrated the promise of using a TLR4 inhibitor such as resatorvid for HCC chemoprevention and suggested that patients with obesity, diabetes and/or NAFLD should be the target population for this chemoprevention approach. IL-6 or IL-10 could be promising markers to further stratify patients to treat.

## MATERIALS AND METHODS

### Mice: tissue collection, treatment and MR Imaging

C57BL/6 mice with hepatocyte-specific *Pten* deletion (Hep*Pten^-^*) were crossed with an Albumin (Alb)-Cre-transgenic mice. For this model, control animals are *Pten*^loxP/loxP^; Alb-Cre^-^ while the experimental mice are *Pten*^loxP/loxP^; Alb-Cre^+^ (Hep*Pten^-^*). All animal procedures were carried out in accordance with the policies and regulations set forth by the Institutional Animal Care and Use Committee (IACUC) at MD Anderson Cancer Center. At necropsy, tumors and liver tissues were harvested and cut into several sections. These sections were either snap-frozen in liquid nitrogen, fixed in 10% neutral formalin, or embedded in OCT in cryomolds for further analysis. Blood samples were also harvested at necropsy and processed for serum and plasma collection for further analysis. For treatment, resatorvid, also called TAK-242 (Calbiochem, MA, United States), was dissolved with DMSO to create a 10 mg/ml stock solution and was further diluted with Dulbecco's Phosphate-Buffered Saline for *in vivo* treatment. A total of nineteen 8 month-old Hep*Pten^-^* male mice were divided into two groups: 1) placebo (*n* = 10) and 2) mice receiving intraperitoneal (IP) injection of resatorvid at a dosage of 10 mg/kg, daily for 28 days (*n* = 9). The Biospec USR47/40 (Bruker Biospin MRI) imaging system was used to image the mice at days 0, 14 and 28. Liver tumors were detected using a standard rapid acquisition with relaxation enhancement (RARE) sequence in the coronal and axial planes with a 250 μm slice thickness and with the number of slices sufficient to cover the entire liver. Tumor slice areas were selected, measured with the ROI manager feature of ImageJ and finally used to calculate tumor volume.

### Histopathological evaluation

Formalin-fixed tissue sections were sectioned and subsequently stained with hematoxylin and eosin (H&E) or Masson's Trichome stains. Histopathological features of the sectioned and stained liver tissues were blindly evaluated by a liver pathologist. The following histopathology parameters were scored: fibrosis (0–4), macrovesicular steatosis (0–3), microvesicular steatosis (0–3), lobular inflammation (0–3), hepatocellular ballooning degeneration (0–2), and bile ductular reaction (0–3).

### Whole-exome sequencing (WES)

DNA was extracted from 20 tumors, distant non tumoral liver and tails collected from 9–12 months male Hep*Pten^-^* mice (*n* = 10) using QIAmp^®^ DNA Mini Kit. Indexed libraries were prepared from Biorupter Ultrasonicator (Diagenode)-sheared gDNA using the KAPA Hyper Library Preparation Kit (Kapa Biosystems). Library quality was assessed using The NGS Fragment Analyzer Reagent (Advanced Analytical Technologies). The libraries were then prepared for capture with 7 cycles of Ligation Mediated PCR (LM-PCR) amplification. Following Ligation Mediated PCR, amplified libraries were assessed for (i) quality using The NGS Fragment Analyzer Reagent (Advanced Analytical Technologies) and (ii) quantity using the Qubit dsDNA HS Assay Kit (ThermoFisher), then multiplexed four libraries per pool. Exome capture was performed using the NimbleGen SeqCap EZ Developer Kit. The enriched libraries were PCR amplified 6 cycles post capture and assessed for the quality using The NGS Fragment Analyzer Reagent (Advanced Analytical Technologies). Enrichment of PCR products was assessed by quantitative PCR and quantified using the Qubit dsDNA HS Assay Kit. Sequencing was performed on the HiSeq4000 Sequencer (Illumina Inc) with four samples per lane using the 76nt paired-end configuration. For Single nucleotide variants (SNVs) and small insertions and deletions (indels) identification, Raw BCL files off the sequencer were processed using Illumina's Consensus Assessment of sequence And Variation (CASAVA) tool (https://support.illumina.com/sequencing/sequencing_software/bcl2fastq-conversion-software.html) for demultiplexing/conversion to FASTQ format. The FASTQ files were aligned to the reference genome (mouse mm10) using BWA [42] with 3 mis-matches with 2 in the first 40 seed regions for the 76 bases of the reads. The aligned BAM files were subjected to mark duplication, re-alignment, and re-calibration using Picard and GATK [43] before any downstream analyses. Somatic mutation calls were determined using MuTect [44] followed by functional annotation using ANNOVAR (http://annovar.openbioinformatics.org/en/latest/). Nonsynonymous SNVs with mutated allele frequency more than 5% and covered by at least 20 reads in tumor and 10 reads in the matching normal, were used for further analysis. Nonsynonymous somatic indels were filtered and selected for further analysis by mutated allele frequency of 5% or more and covered by at least 20 reads in the tumor and 10 reads in the matching normal. SNVs and somatic indels were further filtered for exonic or splicing function. Qiagen's Ingenuity Pathway Analysis software was used for upstream and causal network analysis. The list of genes carrying at least one somatic mutation in tumors of adult male Hep*Pten-* was uploaded and subjected to a core analysis, from which relevant upstream regulators and causal networks were identified.

### Data sources and human subject parameters

The Cancer Genome Atlas (TCGA) HCC data from the cBioPortal online platform (http://www.cbioportal.org/) is the source of human subjects parameters. For TCGA HCC, clinical and demographic information as well as mRNA expression for TLR4, IL-6 and IL-10 from 363 HCCs with available mRNA expression profiling data and after exclusion of fibrolamellar carcinomas, were downloaded from cBioPortal (Supplementary Table 2).

### Statistical analysis

Unpaired Mann-Whitney tests were utilized to assess statistical difference between mice groups. Correlation between TLR4, IL6, and IL10 mRNA expression was evaluated using Spearman rank-order correlation coefficient. In TCGA, we divided the patients into quartiles based on TLR4, IL-6 or IL-10 mRNA expression. For all TCGA analyses, the highest quartiles of expression were compared to the lower three quartiles combined. For crude analysis, we used univariable logistic regression to calculate *p*-values. We then repeated the analyses adjusting for age and gender. All analysis were conducted in SPSS Version 24.0 for Windows. Statistical tests were considered significant at *p* < 0.05.

## SUPPLEMENTARY MATERIALS FIGURES AND TABLES





## Abbreviations

AFP: alpha-feto protein
Alb: albumin
AOR: Adjusted Odds Ratio
APL: Acute Promyelocytic Leukemia
CI: Confidence Interval
CXCL12: C-X-C Motif Chemokine Ligand 12
CXCR4: C-X-C Motif Chemokine Receptor 4
FKBP7: FK506 binding protein 7
FUT7: Fucosyltransferase 7
HBV: Hepatitis B Virus
HCC: Hepatocellular Carcinoma
HCV: Hepatitis C Virus
HepPten-: hepatocyte-specific Pten Deletion
IL10: Interleukin 10
IL10RA: Interleukin 10 Receptor Subunit Alpha
IL6: Interleukin 6
IPA: Ingenuity Pathway Analysis
NAFLD: Non-Alcoholic Fatty Liver Disease
NASH: Non-Alcoholic Steatohepatitis
NKX2-5: NK2 homeobox 5
OR: Odds Ratio
SIGIRR: Single immunoglobulin interleukin-1 receptor related molecule
SNP: Single Nucleotide Polymorphism
SNV: Single Nucleotide Variant
TCGA: The Cancer Genome Atlas
TLR4: Toll-like Receptor 4
WES: Whole-Exome Sequencing
YY1AP1: YY1 Associated Protein 1.
