# Supplementary material for: Toll-like receptor 4: a target for chemoprevention of hepatocellular carcinoma in obesity and steatohepatitis
Source: Oncotarget. 2018 Jun 29;9(50):29495–507. doi: 10.18632/oncotarget.25685 (PMC6047684; doi:10.18632/oncotarget.25685)
Supplement: Supplementary file 1 [file oncotarget-09-29495-s001.pdf]

# Toll-like receptor 4: a target for chemoprevention of hepatocellular carcinoma in obesity and steatohepatitis

## SUPPLEMENTARY MATERIALS

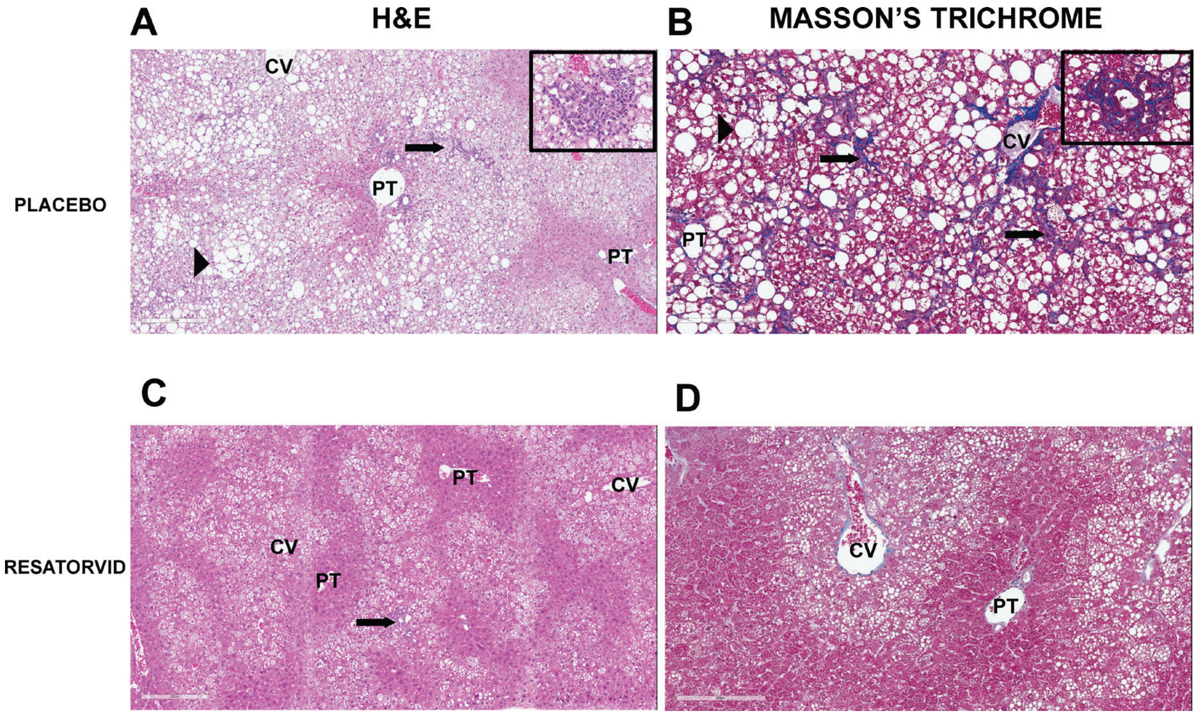


**Supplementary Figure 1: Liver histopathologic changes in HepPten- mice treated with placebo or resatorvid.** Placebo-treated mice (panels **A** and **B**) had significantly more macrovesicular steatosis when compared to resatorvid-treated mice (arrowheads). The placebo-treated group had more steatosis overall, which was often panlobular (i.e., extending from portal tracts to central veins); unlike the resatorvid-treated mice which only had mild perivenular (zone 3) microvesicular steatosis (panels **C** and **D**). In addition, the placebo-treated group had more prominent bile ductular reactions (arrows and A, inset) and increased periportal, subsinusoidal, and perivenular fibrosis (arrows and B, inset). The bile duct lesions often contained scattered inflammatory cells with surrounding fibrosis (A and B, insets). PT = portal tract and CV = central vein.

**Supplementary Table 1: List of somatic mutations detected by whole-exome sequencing in each of the HepPten- mice.** See Supplementary_Table_1

**Supplementary Table 2: Demographic and clinical parameters of 363 HCC patients in TCGA with mRNA expression data for TLR4, IL-6 and IL-10**

| Parameters | Median (range) or frequency (%) |
|---|---|
| **Male** | 244 (67.2%) |
| **Race, Ethnicity** | |
| Asian | 156 (45.3%) |
| White, Non-Hispanic | 155 (45.1%) |
| White, Hispanic | 15 (4.4%) |
| Other | 18 (5.2%) |
| **Age (y)** | 61 (16–90) |
| **Family History of Cancer** | 109 (34.9%) |
| **BMI** | 24.4 (14.5–61.7) |
| **Obese (BMI ≥ 30)** | 66 (20.1%) |
| **NAFLD** | 20 (5.8%) |
| **HBV** | 103 (29.9%) |
| **HCV** | 56 (16.3%) |
| **Alcohol Etiology** | 115 (33.4%) |
| **No known risk factor** | 87 (25.3%) |
| **Cirrhosis** | 95 (33.7%) |
| **Fibrosis** | 161 (64.9%) |
| **Fibrosis Ishak Score** | 24.9 (14.5–56.1) |
| 1, 2 | 35 (22.9%) |
| 3, 4 | 31 (25.3%) |
| 5, 6 | 95 (9.5%) |
| **AFP** (ng/mL) | 16 (1–2035400) |
| **AFP ≥ 20 ng/mL** | 130 (47.6%) |

BMI: body mass index; NAFLD: non-alcoholic fatty liver disease; HBV: hepatitis B virus; HCV: hepatitis C virus; AFP: alpha-fetoprotein.

**Supplementary Table 3: Demographic and clinical variables in 363 HCC patients by tumoral IL-6 mRNA expression**

| | Low IL-6 (Q1–Q3) | High IL-6 (Q4) | *P* | Adjusted OR | *P* |
|---|---|---|---|---|---|
| **IL6 mRNA Expression** (*n* = 363) | 3.19 (0.18)–2.22 | 63.00 (10.24)–22.01 | | | |
| **Male** (*n* = 363) | 187 (68.8%) | 57 (62.6%) | 0.2830 | 0.76 (0.46–1.25) | 0.2794 |
| **Race, Ethnicity** (*n* = 344) | | | <0.0001 | | <0.0001 |
| Asian | 137 (53.3%) | 19 (21.8%) | | REF | |
| White, Non-Hispanic | 102 (39.7%) | 53 (60.9%) | | 4.00 (2.13–7.49) | <0.0001 |
| White, Hispanic | 9 (3.5%) | 6 (6.9%) | | 4.67 (1.48–14.71) | 0.0085 |
| Other | 9 (3.5%) | 9 (10.3%) | | 7.60 (2.67–21.66) | 0.0001 |
| **Diagnosis Age (y)** (*n* = 363) | 59.6 (0.80)–61.0 | 59.5 (1.4)–61.0 | 0.9697 | 0.99 (0.98–1.02) | 0.8910 |
| **Family History of Cancer** (*n* = 312) | 74 (32.0%) | 35 (43.2%) | 0.0707 | 1.59 (0.93–2.72) | 0.0908 |
| **BMI** (*n* = 329) | 25.1 (0.4)–24.0 | 27.7 (0.8)–26.3 | 0.0019 | 1.07 (1.03–1.11) | 0.0018 |
| **NAFLD** (*n* = 344) | 11 (4.3%) | 9 (10.5%) | 0.0392 | 2.59 (1.03–6.52) | 0.0427 |
| **Obese (BMI ≥ 30)** (*n* = 329) | 37 (15.0%) | 29 (35.4%) | 0.0001 | 3.11 (1.74–5.56) | 0.0001 |
| **HBV** (*n* = 344) | 90 (34.9%) | 13 (15.1%) | 0.0008 | 0.34 (0.17–0.65) | 0.0013 |
| **HCV** (*n* = 344) | 32 (12.4%) | 24 (27.9%) | 0.0010 | 2.98 (1.61–5.50) | 0.0005 |
| **Alcohol Etiology** (*n* = 344) | 86 (33.3%) | 29 (33.7%) | 0.9474 | 1.14 (0.66–1.99) | 0.6418 |
| **Cirrhosis** (*n* = 282) | 68 (32.7%) | 27 (36.5%) | 0.5534 | 1.20 (0.68–2.11) | 0.5253 |
| **Fibrosis** (*n* = 248) | 121 (66.5%) | 40 (60.6%) | 0.3919 | 0.82 (0.44–1.50) | 0.5112 |
| **Fibrosis Ishak Score** (*n* = 161) | | | 0.0129 | | 0.0558 |
| 1,2 | 24 (19.8%) | 11 (27.5%) | | REF | |
| 3,4 | 29 (24.0%) | 2 (5.0%) | | 0.15 (0.03–0.76) | 0.0215 |
| 5,6 | 68 (56.2%) | 27 (67.5%) | | 0.89 (0.38–2.07) | 0.7785 |
| **AFP (ng/mL)** (*n* = 273) | 16820 (10277)–17 | 6761 (3646)–11 | 0.6287 | 0.998 (0.99–1.00) | 0.5904 |
| **AFP ≥ 20 ng/mL** (*n* = 273) | 100 (49.5%) | 30 (42.3%) | 0.2934 | 0.70 (0.40–1.23) | 0.2146 |

Data are presented as mean (SEM)-median or as frequency (%)BMI, body mass index; NAFLD, non-alcoholic fatty liver disease; HBV, hepatitis B virus; HCV, hepatitis C virus.

**Supplementary Table 4: Demographic and clinical variables in 363 HCC patients by tumoral IL-10 mRNA expression**

| | Low IL10 (Q1–Q3) | High IL10 (Q4) | *P* | Adjusted OR | *P* |
|---|---|---|---|---|---|
| **IL-10 mRNA Expression** (*n* = 363) | 2.47 (0.11)–2.32 | 18.55 (2.99)–11.05 | | | |
| **Male** (*n* = 363) | 183 (67.3%) | 61 (67.0%) | 0.9654 | 0.96 (0.58–1.60) | 0.8847 |
| **Race, Ethnicity** (*n* = 344) | | | 0.4216 | | 0.535 |
| Asian | 121 (46.9%) | 35 (40.7%) | | REF | |
| White, Non-Hispanic | 116 (45.0%) | 39 (45.3%) | | 1.28 (0.72–2.25) | 0.4009 |
| White, Hispanic | 9 (3.5%) | 6 (7.0%) | | 2.29 (0.76–6.93) | 0.1433 |
| Other | 12 (4.7%) | 6 (7.0%) | | 1.80 (0.63–5.16) | 0.275 |
| **Diagnosis Age (y)** (*n* = 363) | 60.0 (0.8)–61.0 | 58.1 (1.4)–59.0 | 0.2367 | 0.99 (0.97–1.01) | 0.2332 |
| **Family History of Cancer** (*n* = 312) | 75 (32.5%) | 34 (42.0%) | 0.1237 | 1.63 (0.95–2.79) | 0.0785 |
| **BMI** (*n* = 329) | 25.3 (0.4)–24.2 | 27.1 (0.9)–25.0 | 0.0242 | 1.05 (1.01–1.10) | 0.0153 |
| **NAFLD** (*n* = 344) | 14 (5.4%) | 6 (7.0%) | 0.5956 | 1.33 (0.49–3.59) | 0.5726 |
| **Obese (BMI ≥ 30)** (*n* = 329) | 41 (16.5%) | 25 (30.9%) | 0.0059 | 2.34 (1.30–4.23) | 0.0046 |
| **HBV** (*n* = 344) | 83 (32.2%) | 20 (23.3%) | 0.1198 | 0.59 (0.33–1.06) | 0.0783 |
| **HCV** (*n* = 344) | 41 (15.95) | 15 (17.4%) | 0.736 | 1.14 (0.59–2.19) | 0.7044 |
| **Alcohol Etiology** (*n* = 344) | 80 (31.0%) | 35 (40.7%) | 0.1002 | 1.69 (0.98–2.92) | 0.0593 |
| **Cirrhosis** (*n* = 282**)** | 75 (34.7%) | 20 (30.3%) | 0.5066 | 0.82 (0.45–1.50) | 0.517 |
| **Fibrosis** (*n* = 248) | 125 (64.4%) | 36 (66.7%) | 0.761 | 1.26 (0.64–2.46) | 0.5087 |
| **Fibrosis Ishak Score** (*n* = 161) | | | 0.3057 | | 0.4422 |
| 1, 2 | 24 (19.2%) | 11 (30.6%) | REF | REF | |
| 3, 4 | 26 (20.8%) | 5 (13.9%) | | 0.40 (0.12–1.33) | 0.1328 |
| 5, 6 | 75 (60.0%) | 20 (55.6%) | | 0.55 (0.23–1.32) | 0.1818 |
| **AFP (ng/mL)** (*n* = 273) | 5077 (1838)–11 | 42829 (31081)–25 | 0.1336 | 1.00 (1.00–1.00) | 0.1422 |
| **AFP ≥ 20 ng/mL** (*n* = 273) | 93 (44.9%) | 37 (56.1%) | 0.1162 | 1.52 (0.86–2.67) | 0.1491 |

Data are presented as mean (SEM)-median or as frequency (%). BMI, body mass index; NAFLD, non-alcoholic fatty liver disease; HBV, hepatitis B virus; HCV, hepatitis C virus.
