# Supplementary material for: Toll-like receptor 4: a target for chemoprevention of hepatocellular carcinoma in obesity and steatohepatitis
Source: Oncotarget. 2018 Jun 29;9(50):29495–507. doi: 10.18632/oncotarget.25685 (PMC6047684; doi:10.18632/oncotarget.25685)
Supplement: Supplementary file 2 [file oncotarget-09-29495-s002.docx]

**Supplementary Table 1. List of somatic mutations detected by whole-exome sequencing in each of the Hep*Pten*^-^ mice.** T: tumor ID; Ref_allele: base in the reference genome; Alt_allele: base altered in the sample; Chr.: chromosome; Exonic func: Function of exonic mutation; SNV: single nucleotide variants; Aaannotation: amino acid change; Tumor_f: allele frequency

| **Mouse ID** | **T** | **Gene** | **Start_**  **position** | **Ref_**  **allele** | **Alt_**  **allele** | **Chr.** | **Exonic**  **func** | **Aaannotation** | **Number of mice with this mutation** | **Present also in adjacent liver** | **Tumor_f** |
| --- | --- | --- | --- | --- | --- | --- | --- | --- | --- | --- | --- |
| 3559 | T1 | Slc9a3r1 | 115163538 | G | A | 11 | nonsynonymous SNV | p.A5T | 1 |  | 0.11 |
| 3559 | T1 | Slc17a7 | 45173434 | G | A | 7 | nonsynonymous SNV | p.G445D | 1 |  | 0.13 |
| 3559 | T1 | Amt | 108299441 | C | T | 9 | nonsynonymous SNV | p.A178V | 1 |  | 0.08 |
| 3559 | T1 | Agxt2 | 10377393 | NN | N | 15 | frameshift deletion | p.A126fs | 1 |  | 0.08 |
| 3629 | T1 | 9130409I23Rik | 181055147 | G | A | 1 | nonsynonymous SNV | p.R158K | 1 |  | 0.11 |
| 3629 | T1 | Ptdss1 | 66995383 | G | A | 13 | nonsynonymous SNV | p.E427K | 1 |  | 0.16 |
| 3629 | T1 | Prex1 | 166581906 | C | A | 2 | splicing |  | 1 |  | 0.07 |
| 3629 | T1 | Swap70 | 110255778 | G | T | 7 | nonsynonymous SNV | p.W95L | 1 |  | 0.10 |
| 3629 | T1 | Mettl7a1 | 100313085 | TC | T | 15 | frameshift deletion | p.L197fs | 1 |  | 0.05 |
| 3629 | T1 | Fgd4 | 16477515 | T | TA | 16 | splicing |  | 1 |  | 0.07 |
| 3629 | T1 | Spice1 | 44386790 | NN | N | 16 | frameshift deletion | p.E845fs | 2 | yes | 0.09 |
| 3629 | T2 | Esco2 | 65831404 | A | T | 14 | nonsynonymous SNV | p.S152R | 1 |  | 0.13 |
| 3629 | T2 | Asb8 | 98135894 | C | T | 15 | nonsynonymous SNV | p.R260H | 1 |  | 0.13 |
| 3629 | T2 | Otud1 | 19658809 | G | A | 2 | nonsynonymous SNV | p.A250T | 1 |  | 0.09 |
| 3629 | T2 | Ttn | 76751879 | T | C | 2 | nonsynonymous SNV | p.K21144R | 1 |  | 0.08 |
| 3629 | T2 | Kdm2b | 122932751 | C | T | 5 | nonsynonymous SNV | p.D428N | 1 |  | 0.17 |
| 3629 | T2 | Asic3 | 24416955 | G | A | 5 | nonsynonymous SNV | p.G355E | 1 |  | 0.11 |
| 3629 | T2 | Adam20 | 40796517 | G | T | 8 | nonsynonymous SNV | p.G555C | 1 |  | 0.12 |
| 3629 | T2 | Grb10 | 11987676 | G | GA | 11 | splicing |  | 2 |  | 0.11 |
| 3629 | T2 | Fkbp7 | 76671673 | G | GA | 2 | frameshift insertion | p.A129fs | 2 |  | 0.06 |
| 3629 | T2 | Scn1b | 31125203 | TT | T | 7 | frameshift deletion | p.G20fs | 1 |  | 0.12 |
| 3629 | T3 | Serpinb3a | 107047565 | T | G | 1 | nonsynonymous SNV | p.M171L | 2 |  | 0.06 |
| 3629 | T3 | Serpinb3d | 107078537 | T | A | 1 | nonsynonymous SNV | p.M274L | 2 |  | 0.13 |
| 3629 | T3 | Serpinb3b | 107154379 | C | G | 1 | nonsynonymous SNV | p.C385S | 2 |  | 0.06 |
| 3629 | T3 | Serpinb3b | 107155886 | T | C | 1 | nonsynonymous SNV | p.N188D | 2 |  | 0.09 |
| 3629 | T3 | Cdh7 | 110065735 | C | G | 1 | nonsynonymous SNV | p.L307V | 2 |  | 0.10 |
| 3629 | T3 | Zranb3 | 127960096 | T | G | 1 | nonsynonymous SNV | p.I869L | 1 |  | 0.07 |
| 3629 | T3 | Ubxn4 | 128258983 | C | T | 1 | nonsynonymous SNV | p.T162I | 1 |  | 0.14 |
| 3629 | T3 | Ikbke | 131265937 | C | A | 1 | nonsynonymous SNV | p.A483S | 1 |  | 0.18 |
| 3629 | T3 | Ikbke | 131269823 | T | C | 1 | nonsynonymous SNV | p.S471G | 1 |  | 0.13 |
| 3629 | T3 | Fam72a | 131530668 | T | C | 1 | nonsynonymous SNV | p.I56T | 1 |  | 0.10 |
| 3629 | T3 | Ctse | 131672306 | G | T | 1 | nonsynonymous SNV | p.Q297H | 1 |  | 0.07 |
| 3629 | T3 | Slc26a9 | 131763211 | A | G | 1 | nonsynonymous SNV | p.T576A | 1 |  | 0.13 |
| 3629 | T3 | Rab29 | 131872110 | A | G | 1 | nonsynonymous SNV | p.Q141R | 1 |  | 0.13 |
| 3629 | T3 | Mdm4 | 132991702 | G | A | 1 | nonsynonymous SNV | p.A476V | 1 |  | 0.15 |
| 3629 | T3 | Mdm4 | 133009145 | A | T | 1 | nonsynonymous SNV | p.I113N | 1 |  | 0.10 |
| 3629 | T3 | Ccdc112 | 46296325 | T | C | 18 | nonsynonymous SNV | p.E102G | 1 |  | 0.05 |
| 3629 | T3 | Car2 | 14887916 | G | C | 3 | nonsynonymous SNV | p.Q39H | 1 |  | 0.14 |
| 3629 | T3 | Ifna5 | 88835982 | A | T | 4 | nonsynonymous SNV | p.Y153F | 1 |  | 0.06 |
| 3629 | T3 | Itgb1 | 128705426 | T | A | 8 | nonsynonymous SNV | p.L5Q | 1 |  | 0.07 |
| 3629 | T3 | 1700008F21Rik | 129163852 | T | G | 8 | nonsynonymous SNV | p.L272V | 1 |  | 0.07 |
| 3629 | T3 | 5330429B09Rik | 19785033 | G | C | 8 | nonsynonymous SNV | p.W96C | 1 |  | 0.07 |
| 3629 | T3 | Srgap2 | 131294988 | N | NA | 1 | frameshift insertion | p.F270fs | 1 |  | 0.12 |
| 3629 | T3 | Esco2 | 65831412 | C | CT | 14 | frameshift insertion | p.Q150fs | 1 |  | 0.05 |
| 3629 | T3 | Mdc1 | 35847894 | GTGA | G | 17 | nonframeshift deletion | p.389_389del | 2 |  | 0.08 |
| 3629 | T3 | Gimap4 | 48691372 | TC | T | 6 | frameshift deletion | p.T51fs | 1 |  | 0.06 |
| 3631 | T1 | Mdc1 | 35847894 | GTGA | G | 17 | nonframeshift deletion | p.389_389del | 2 |  | 0.07 |
| 3631 | T1 | Dennd4b | 90275569 | CAAG | C | 3 | nonframeshift deletion | p.909_909del | 1 |  | 0.08 |
| 3694 | T1 | Fkbp7 | 76671673 | G | GA | 2 | frameshift insertion | p.A129fs | 2 |  | 0.07 |
| 3694 | T1 | Fam13a | 58939350 | N | NT | 6 | frameshift insertion | p.M634fs | 1 |  | 0.06 |
| 3694 | T2 | Actr2 | 20094375 | C | T | 11 | nonsynonymous SNV | p.G77D | 1 |  | 0.08 |
| 3694 | T2 | Hand1 | 57831456 | T | A | 11 | nonsynonymous SNV | p.N111Y | 1 |  | 0.25 |
| 3694 | T2 | Nf2 | 4772640 | T | TA | 11 | splicing |  | 1 |  | 0.14 |
| 3694 | T2 | Ryr2 | 11568552 | N | NA | 13 | frameshift insertion | p.A4739fs | 1 |  | 0.10 |
| 3694 | T2 | Lmo7 | 101933987 | CT | C | 14 | nonframeshift deletion | p.X1454X | 1 | yes | 0.14 |
| 3694 | T2 | Adgrf2 | 42719493 | TT | T | 17 | splicing |  | 1 |  | 0.06 |
| 3694 | T2 | Fkbp7 | 76671673 | G | GA | 2 | frameshift insertion | p.A129fs | 2 |  | 0.05 |
| 3694 | T2 | Cyp2s1 | 25816443 | CCTA | C | 7 | nonframeshift deletion | p.20_20del | 1 |  | 0.10 |
| 3694 | T2 | Senp6 | 80136750 | AT | A | 9 | frameshift deletion | p.K888fs | 1 |  | 0.10 |
| 3700 | T1 | Zzef1 | 72864757 | G | A | 11 | nonsynonymous SNV | p.S1004N | 1 |  | 0.07 |
| 3700 | T1 | Ston2 | 91648538 | G | T | 12 | nonsynonymous SNV | p.S365R | 1 |  | 0.11 |
| 3700 | T1 | Syt10 | 89827077 | C | T | 15 | stopgain | p.W84X | 1 |  | 0.07 |
| 3700 | T1 | Atp5g3 | 73909200 | T | C | 2 | nonsynonymous SNV | p.I99V | 1 | yes | 0.08 |
| 3700 | T1 | Speer4a | 26038248 | T | C | 5 | nonsynonymous SNV | p.K80E | 1 |  | 0.18 |
| 3700 | T1 | Capn3 | 120498973 | T | TA | 2 | frameshift insertion | p.K596fs | 1 |  | 0.10 |
| 3700 | T1 | Atxn2l | 126494249 | CAGC | C | 7 | nonframeshift deletion | p.778_779del | 1 |  | 0.11 |
| 3700 | T2 | Dux | 58232231 | T | G | 10 | nonsynonymous SNV | p.T149P | 1 |  | 0.05 |
| 3700 | T2 | Zzef1 | 72864757 | G | A | 11 | nonsynonymous SNV | p.S1004N | 1 |  | 0.10 |
| 3700 | T2 | Add3 | 53245263 | C | A | 19 | nonsynonymous SNV | p.N698K | 1 |  | 0.08 |
| 3700 | T2 | Atp5g3 | 73909200 | T | C | 2 | nonsynonymous SNV | p.I99V | 1 | yes | 0.08 |
| 3700 | T2 | Lemd3 | 120978272 | TCAC | T | 10 | nonframeshift deletion | p.351_352del | 1 |  | 0.05 |
| 3700 | T2 | Akap7 | 25283990 | AATT | A | 10 | nonframeshift deletion | p.30_31del | 1 |  | 0.06 |
| 3700 | T2 | Fbxw10 | 62850809 | A | AA | 11 | frameshift insertion | p.L251fs | 1 |  | 0.09 |
| 3700 | T2 | Ccdc88b | 6857712 | CTGT | C | 19 | nonframeshift deletion | p.46_47del | 1 |  | 0.06 |
| 3700 | T2 | Cyp2u1 | 131293440 | C | CAC | 3 | frameshift insertion | p.V497fs | 1 |  | 0.05 |
| 3700 | T2 | Aldob | 49544920 | A | AA | 4 | splicing |  | 1 |  | 0.13 |
| 3700 | T3 | Polr2a | 69736791 | C | T | 11 | nonsynonymous SNV | p.C1430Y | 1 |  | 0.08 |
| 3700 | T3 | Pkhd1 | 20201111 | C | T | 1 | nonsynonymous SNV | p.A3073T | 1 |  | 0.09 |
| 3700 | T3 | Rbm34 | 126965527 | G | A | 8 | nonsynonymous SNV | p.A155V | 1 |  | 0.10 |
| 3700 | T3 | Best3 | 116992528 | TC | T | 10 | frameshift deletion | p.Q58fs | 1 |  | 0.06 |
| 3700 | T3 | Impg2 | 56257940 | CCTG | C | 16 | nonframeshift deletion | p.422_422del | 1 |  | 0.05 |
| 3700 | T3 | Nhlrc3 | 53462298 | TA | T | 3 | frameshift deletion | p.H84fs | 1 |  | 0.05 |
| 3700 | T3 | Scn11a | 119779020 | TA | T | 9 | frameshift deletion | p.K1015fs | 1 |  | 0.13 |
| 3711 | T1 | Smpx | 157702954 | A | T | X | splicing |  | 1 |  | 0.18 |
| 3711 | T1 | Spice1 | 44386790 | NN | N | 16 | frameshift deletion | p.E845fs | 2 |  | 0.08 |
| 3711 | T1 | Polr3c | 96719204 | TT | T | 3 | frameshift deletion | p.G163fs | 2 |  | 0.06 |
| 3711 | T1 | Trim63 | 134327707 | CTAC | C | 4 | nonframeshift deletion | p.328_328del | 1 |  | 0.13 |
| 3711 | T2 | Gopc | 52381751 | C | G | 10 | nonsynonymous SNV | p.D50H | 1 |  | 0.06 |
| 3711 | T2 | Selp | 164148495 | G | A | 1 | nonsynonymous SNV | p.V758I | 1 |  | 0.21 |
| 3711 | T2 | AI607873 | 173729382 | A | G | 1 | nonsynonymous SNV | p.Y604H | 1 |  | 0.12 |
| 3711 | T2 | Xylt2 | 94670065 | C | T | 11 | nonsynonymous SNV | p.V216I | 1 |  | 0.36 |
| 3711 | T2 | Pcdh7 | 57720338 | G | A | 5 | nonsynonymous SNV | p.V412I | 1 |  | 0.14 |
| 3711 | T2 | Dennd2a | 39483087 | C | T | 6 | nonsynonymous SNV | p.V695I | 1 |  | 0.22 |
| 3711 | T2 | Foxc1 | 31807996 | TTAT | T | 13 | nonframeshift deletion | p.263_264del | 1 |  | 0.08 |
| 3711 | T2 | Calm5 | 3854680 | NN | N | 13 | frameshift deletion | p.F125fs | 1 |  | 0.13 |
| 3711 | T2 | Nop58 | 59711286 | AGCATTA | A | 1 | nonframeshift deletion | p.526_527del | 1 |  | 0.17 |
| 3711 | T2 | Mylk | 35000506 | AAAA | A | 16 | nonframeshift deletion | p.1943_1943del | 1 |  | 0.17 |
| 3711 | T2 | Cep89 | 35409643 | TTAA | T | 7 | nonframeshift deletion | p.182_183del | 1 |  | 0.07 |
| 3711 | T2 | Nxpe4 | 48396754 | N | NA | 9 | frameshift insertion | p.R386fs | 1 |  | 0.11 |
| 3711 | T3 | Gopc | 52381751 | C | G | 10 | nonsynonymous SNV | p.D50H | 1 |  | 0.09 |
| 3711 | T3 | Xylt2 | 94670065 | C | T | 11 | nonsynonymous SNV | p.V216I | 1 |  | 0.32 |
| 3711 | T3 | Selplg | 113819755 | C | G | 5 | nonsynonymous SNV | p.E163D | 1 |  | 0.07 |
| 3711 | T3 | Dennd2a | 39483087 | C | T | 6 | nonsynonymous SNV | p.V695I | 1 |  | 0.30 |
| 3711 | T3 | Hdx | 111659923 | G | T | X | nonsynonymous SNV | p.T323K | 1 |  | 0.09 |
| 3711 | T3 | Ankrd44 | 54729876 | G | GA | 1 | splicing |  | 1 | yes | 0.13 |
| 3711 | T3 | Nop58 | 59711286 | AGCATTA | A | 1 | nonframeshift deletion | p.526_527del | 1 |  | 0.19 |
| 3711 | T3 | Klhl22 | 17784267 | TGA | T | 16 | frameshift deletion | p.C393fs | 1 |  | 0.08 |
| 3711 | T3 | Syngap1 | 26970583 | TC | T | 17 | frameshift deletion | p.P1321fs | 1 |  | 0.05 |
| 3711 | T3 | Tcf7l2 | 55931436 | G | GA | 19 | frameshift insertion | p.R459fs | 1 |  | 0.11 |
| 3711 | T3 | Ankrd60 | 173572470 | G | GA | 2 | frameshift insertion | p.Q169fs | 1 |  | 0.07 |
| 3711 | T3 | Ehmt1 | 24863931 | C | CA | 2 | splicing |  | 1 |  | 0.08 |
| 3711 | T3 | Mdk | 91931373 | CT | C | 2 | frameshift deletion | p.K25fs | 1 |  | 0.14 |
| 3711 | T3 | Tmprss11b | 86660628 | C | CA | 5 | splicing |  | 1 |  | 0.12 |
| 3920 | T1 | Proser1 | 53472019 | C | T | 3 | nonsynonymous SNV | p.S138L | 1 |  | 0.13 |
| 3920 | T1 | Hras | 141192551 | G | T | 7 | nonsynonymous SNV | p.Q61K | 1 |  | 0.19 |
| 3920 | T1 | Ube2dnl1 | 114905467 | G | T | X | nonsynonymous SNV | p.R13L | 1 |  | 0.10 |
| 3920 | T1 | Yy1 | 108793539 | ACAT | A | 12 | nonframeshift deletion | p.43_43del | 1 |  | 0.05 |
| 3920 | T1 | Cntrl | 35127512 | T | TA | 2 | frameshift insertion | p.E444fs | 1 |  | 0.10 |
| 3920 | T1 | C530008M17Rik | 76841862 | T | TA | 5 | frameshift insertion | p.E125fs | 1 |  | 0.07 |
| 3920 | T1 | Chd2 | 73455774 | CT | C | 7 | frameshift deletion | p.K1245fs | 1 |  | 0.06 |
| 3920 | T1 | Senp6 | 80136749 | A | AA | 9 | frameshift insertion | p.M887fs | 1 |  | 0.11 |
| 3920 | T2 | Kcnh8 | 52978053 | C | G | 17 | nonsynonymous SNV | p.P1017R | 1 |  | 0.09 |
| 3920 | T2 | Map1s | 70914145 | C | T | 8 | nonsynonymous SNV | p.P565S | 1 |  | 0.10 |
| 3920 | T2 | Casc3 | 98831395 | A | AC | 11 | frameshift insertion | p.K686fs | 1 |  | 0.05 |
| 3920 | T2 | Tep1 | 50854250 | A | AT | 14 | frameshift insertion | p.N611fs | 1 |  | 0.07 |
| 3920 | T2 | Rnf168 | 32285445 | CACA | C | 16 | nonframeshift deletion | p.154_154del | 1 |  | 0.07 |
| 3920 | T2 | Polr3c | 96719204 | TT | T | 3 | frameshift deletion | p.G163fs | 2 |  | 0.06 |
| 3943 | T1 | Mtbp | 55567284 | C | T | 15 | nonsynonymous SNV | p.R246W | 1 |  | 0.06 |
| 3943 | T1 | Gm9758 | 14914692 | C | G | 5 | nonsynonymous SNV | p.W46C | 1 |  | 0.06 |
| 3943 | T1 | Ccdc170 | 4561012 | ATCT | A | 10 | nonframeshift deletion | p.684_685del | 1 |  | 0.12 |
| 3943 | T1 | Ylpm1 | 85044471 | CA | C | 12 | frameshift deletion | p.K1810fs | 1 |  | 0.05 |
| 3943 | T1 | Hs6st3 | 119138708 |  |  | 14 | nonframeshift deletion | p.98_99del | 1 |  | 0.09 |
| 3943 | T1 | Plcl1 | 55696682 | AG | A | 1 | frameshift deletion | p.Q394fs | 1 |  | 0.13 |
| 3943 | T1 | Stxbp3a | 108825894 | A | AA | 3 | frameshift insertion | p.P124fs | 2 |  | 0.19 |
| 3943 | T1 | Tmprss11a | 86431861 | AT | A | 5 | frameshift deletion | p.N85fs | 1 |  | 0.08 |
| 3962 | T1 | Zfp808 | 62171329 | A | G | 13 | nonsynonymous SNV | p.K124R | 1 |  | 0.16 |
| 3962 | T1 | Mroh2a | 88235216 | G | T | 1 | nonsynonymous SNV | p.Q357H | 1 | yes | 0.19 |
| 3962 | T1 | Slc12a9 | 137323123 | C | T | 5 | nonsynonymous SNV | p.V423I | 1 |  | 0.10 |
| 3962 | T1 | Dhx38 | 109558951 | A | T | 8 | nonsynonymous SNV | p.F417I | 1 |  | 0.21 |
| 3962 | T1 | Mon2 | 123036138 | T | TA | 10 | splicing |  | 1 |  | 0.05 |
| 3962 | T1 | Smarcc2 | 128487618 | AC | A | 10 | frameshift deletion | p.P1072fs | 1 |  | 0.14 |
| 3962 | T1 | Colgalt2 | 152471783 | TG | T | 1 | frameshift deletion | p.E156fs | 1 |  | 0.08 |
| 3962 | T1 | Pfas | 69003874 | C | CA | 11 | splicing |  | 1 | yes | 0.09 |
| 3962 | T1 | Zfp318 | 46412515 | TTAA | T | 17 | nonframeshift deletion | p.1815_1815del | 1 |  | 0.05 |
| 3962 | T1 | Cops7b | 86605142 | CA | C | 1 | frameshift deletion | p.P241fs | 1 |  | 0.06 |
| 3962 | T1 | Brpf1 | 113320540 | TCCA | T | 6 | nonframeshift deletion | p.1007_1008del | 1 |  | 0.14 |
| 3962 | T1 | Ankrd42 | 92584495 | AC | A | 7 | frameshift deletion | p.K505fs | 1 |  | 0.06 |
| 4043 | T1 | Gm1995 | 87863075 | A | G | 12 | nonsynonymous SNV | p.T571A | 1 | yes | 0.21 |
| 4043 | T1 | Dopey2 | 93747549 | C | G | 16 | nonsynonymous SNV | p.P193A | 1 |  | 0.14 |
| 4043 | T1 | Fndc3a | 72599020 | AGT | A | 14 | frameshift deletion | p.V99fs | 1 |  | 0.15 |
| 4043 | T1 | Ctcf | 105664534 | CC | C | 8 | frameshift deletion | p.K258fs | 1 |  | 0.07 |
| 4043 | T1 | Camkv | 107946603 | A | AT | 9 | frameshift insertion | p.T12fs | 1 |  | 0.21 |
| 4043 | T2 | Gm1995 | 87863075 | A | G | 12 | nonsynonymous SNV | p.T571A | 1 | yes | 0.08 |
| 4043 | T2 | Krt78 | 101947046 | T | C | 15 | nonsynonymous SNV | p.M777V | 1 |  | 0.11 |
| 4043 | T2 | Vmn2r101 | 19589825 | T | C | 17 | nonsynonymous SNV | p.L291S | 1 |  | 0.21 |
| 4043 | T2 | Gm20939 | 94876861 | G | T | 17 | nonsynonymous SNV | p.E313D | 1 |  | 0.13 |
| 4043 | T2 | Prdm2 | 143135752 | G | C | 4 | nonsynonymous SNV | p.P323A | 1 |  | 0.11 |
| 4043 | T2 | Kctd19 | 105408542 | C | T | 8 | nonsynonymous SNV | p.S29N | 1 |  | 0.09 |
| 4043 | T2 | Gns | 121371269 | TC | T | 10 | frameshift deletion | p.D98fs | 1 |  | 0.07 |
| 4043 | T2 | Hdac5 | 102202147 | TTGA | T | 11 | nonframeshift deletion | p.605_606del | 1 |  | 0.08 |
| 4043 | T2 | Grb10 | 11987676 | G | GA | 11 | splicing |  | 2 |  | 0.14 |
| 4043 | T2 | Fndc3a | 72599020 | AGT | A | 14 | frameshift deletion | p.V99fs | 1 |  | 0.13 |
| 4043 | T2 | Gigyf2 | 87443797 | GGTT | G | 1 | nonframeshift deletion | p.1211_1211del | 1 |  | 0.11 |
| 4043 | T2 | Rtn3 | 7482995 | ATCA | A | 19 | nonframeshift deletion | p.29_30del | 1 |  | 0.09 |
| 4043 | T2 | Fam83d | 158779918 | AA | A | 2 | frameshift deletion | p.R183fs | 1 |  | 0.07 |
| 4043 | T2 | Kmt2a | 44881102 | TT | T | 9 | frameshift deletion | p.P58fs | 1 |  | 0.13 |
| 4043 | T3 | Serpinb3a | 107047565 | T | G | 1 | nonsynonymous SNV | p.M171L | 2 |  | 0.09 |
| 4043 | T3 | Serpinb3d | 107078537 | T | A | 1 | nonsynonymous SNV | p.M274L | 2 |  | 0.12 |
| 4043 | T3 | Serpinb3b | 107154379 | C | G | 1 | nonsynonymous SNV | p.C385S | 2 |  | 0.08 |
| 4043 | T3 | Serpinb3b | 107155886 | T | C | 1 | nonsynonymous SNV | p.N188D | 2 |  | 0.08 |
| 4043 | T3 | Serpinb3c | 107273171 | A | G | 1 | nonsynonymous SNV | p.V172A | 1 |  | 0.07 |
| 4043 | T3 | Serpinb3c | 107274993 | T | C | 1 | nonsynonymous SNV | p.D95G | 1 |  | 0.09 |
| 4043 | T3 | Cdh7 | 110065735 | C | G | 1 | nonsynonymous SNV | p.L307V | 2 |  | 0.13 |
| 4043 | T3 | Cdh19 | 110893384 | C | A | 1 | nonsynonymous SNV | p.E541D | 1 |  | 0.11 |
| 4043 | T3 | Prmt5 | 54514826 | G | A | 14 | nonsynonymous SNV | p.A171V | 1 |  | 0.07 |
| 4043 | T3 | Tsc22d1 | 76417122 | C | A | 14 | nonsynonymous SNV | p.A347D | 1 |  | 0.09 |
| 4043 | T3 | Birc6 | 74599330 | A | G | 17 | nonsynonymous SNV | p.I1405M | 1 |  | 0.07 |
| 4043 | T3 | Rrp12 | 41886101 | C | T | 19 | nonsynonymous SNV | p.A369T | 1 |  | 0.12 |
| 4043 | T3 | Ube2u | 100486704 | A | G | 4 | nonsynonymous SNV | p.N100S | 1 |  | 0.07 |
| 4043 | T3 | Dnajc6 | 101616817 | C | A | 4 | nonsynonymous SNV | p.P472T | 1 |  | 0.09 |
| 4043 | T3 | Leprot | 101656291 | A | G | 4 | nonsynonymous SNV | p.D92G | 1 |  | 0.07 |
| 4043 | T3 | Lepr | 101764947 | A | G | 4 | nonsynonymous SNV | p.I359V | 1 |  | 0.11 |
| 4043 | T3 | 4921539E11Rik | 103231220 | C | T | 4 | nonsynonymous SNV | p.R364H | 1 |  | 0.09 |
| 4043 | T3 | 4921539E11Rik | 103231253 | C | T | 4 | nonsynonymous SNV | p.R353H | 1 |  | 0.06 |
| 4043 | T3 | 4921539E11Rik | 103231373 | A | G | 4 | nonsynonymous SNV | p.V313A | 1 |  | 0.17 |
| 4043 | T3 | C87977 | 144207557 | A | C | 4 | nonsynonymous SNV | p.L327V | 1 |  | 0.06 |
| 4043 | T3 | Atg4c | 99221365 | G | A | 4 | nonsynonymous SNV | p.A190T | 1 |  | 0.10 |
| 4043 | T3 | Pign | 105646679 | T | TA | 1 | frameshift insertion | p.C314fs | 1 |  | 0.10 |
| 4043 | T3 | Vps13a | 16780769 | TT | T | 19 | frameshift deletion | p.M1fs | 1 |  | 0.11 |
| 4043 | T3 | Stxbp3a | 108825894 | A | AA | 3 | frameshift insertion | p.P124fs | 2 | yes | 0.13 |
